# Opportunities for Enhanced Strategic Use of Surveys, Medical Records, and Program Data for HIV Surveillance of Key Populations: Scoping Review

**DOI:** 10.2196/publichealth.8042

**Published:** 2018-05-22

**Authors:** Sharon Stucker Weir, Stefan D Baral, Jessie K Edwards, Sabrina Zadrozny, James Hargreaves, Jinkou Zhao, Keith Sabin

**Affiliations:** ^1^ Carolina Population Center Department of Epidemiology University of North Carolina Chapel Hill, NC United States; ^2^ Center for Public Health and Human Rights Department of Epidemiology Johns Hopkins School of Public Health Baltimore, MD United States; ^3^ Department of Epidemiology School of Public Health University of North Carolina Chapel Hill, NC United States; ^4^ Carolina Population Center University of North Carolina Chapel Hill, NC United States; ^5^ Department of Social and Environmental Health London School of Hygiene and Tropical Medicine London United Kingdom; ^6^ Technical.Advice and Partnerships Department The Global Fund to Fight AIDS, Tuberculosis and Malaria Geneva Switzerland; ^7^ Joint United Nations Programme on HIV/AIDS Geneva Switzerland

**Keywords:** HIV, sex workers, epidemiology

## Abstract

**Background:**

Normative guidelines from the World Health Organization recommend tracking strategic information indicators among key populations. Monitoring progress in the global response to the HIV epidemic uses indicators put forward by the Joint United Nations Programme on HIV/AIDS. These include the 90-90-90 targets that require a realignment of surveillance data, routinely collected program data, and medical record data, which historically have developed separately.

**Objective:**

The aim of this study was to describe current challenges for monitoring HIV-related strategic information indicators among key populations ((men who have sex with men [MSM], people in prisons and other closed settings, people who inject drugs, sex workers, and transgender people) and identify future opportunities to enhance the use of surveillance data, programmatic data, and medical record data to describe the HIV epidemic among key populations and measure the coverage of HIV prevention, care, and treatment programs.

**Methods:**

To provide a historical perspective, we completed a scoping review of the expansion of HIV surveillance among key populations over the past three decades. To describe current efforts, we conducted a review of the literature to identify published examples of SI indicator estimates among key populations. To describe anticipated challenges and future opportunities to improve measurement of strategic information indicators, particularly from routine program and health data, we consulted participants of the Third Global HIV Surveillance Meeting in Bangkok, where the 2015 World Health Organization strategic information guidelines were launched.

**Results:**

There remains suboptimal alignment of surveillance and programmatic data, as well as routinely collected medical records to facilitate the reporting of the 90-90-90 indicators for HIV among key populations. Studies (n=3) with estimates of all three 90-90-90 indicators rely on cross-sectional survey data. Programmatic data and medical record data continue to be insufficiently robust to provide estimates of the 90-90-90 targets for key populations.

**Conclusions:**

Current reliance on more active data collection processes, including key population-specific surveys, remains warranted until the quality and validity of passively collected routine program and medical record data for key populations is optimized.

## Introduction

### New Vision for Strategic Information

**Figure 1:**
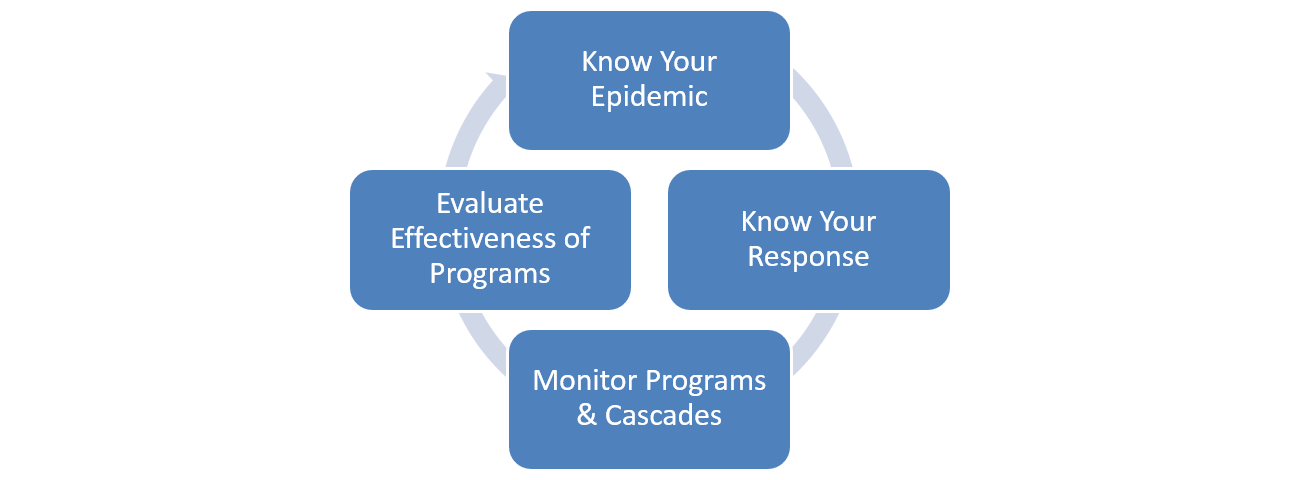
Strategic information cycle.

The 2015 World Health Organization (WHO) guidelines for HIV-related strategic information (SI) [1] present a consolidated framework across the full information cycle for HIV (Figure 1), from traditional surveillance indicators, such as HIV prevalence, incidence, and geographic distribution, to indicators monitoring program response, target setting, coverage, and effectiveness. To provide an integrated assessment of progress along one HIV result chain, the guidelines recommend aligning data elements from multiple sources, including health facilities, HIV prevention and treatment programs, and population-based surveys, and disaggregating indicators by population groups. See Figure 1.

Disaggregation of HIV surveillance indicators [2] for the five key populations recognized by WHO (men who have sex with men [MSM], people in prisons and other closed settings, people who inject drugs, sex workers, and transgender people [3]) is an important objective, given that key populations and their sexual partners accounted for about 45% of all new HIV infections in 2015 [4]. Additionally, the role of key populations within the HIV epidemics in countries with generalized epidemics may be greater than previously thought [5,6]. Stigma and social and economic vulnerabilities pose sustained challenges for the uptake of HIV prevention and treatment services among key populations, making it especially important to document gaps in program coverage for these populations [7,8].

### Operational Challenges for Indicators for Key Populations

The guidelines recommend estimating key population-specific indicators drawn from multiple data sources; however, the operational challenges of this are significant. First, there are often no standardized definitions for key populations and subgroups. Transgender women are not consistently classified separately from MSM; women who exchange sex for goods or services or work part-time may or may not be included as sex workers; and MSM are often considered one group, despite the significant diversity of HIV acquisition and transmission risks among MSM [9,10]. Eligibility criteria for participation in surveys are generally more restrictive than for programs. Sex worker surveys may restrict participation to women aged 18 years and older who report commercial sex in the past 3 months, whereas programs do not generally screen out part-time sex workers or adolescents who exchange sex for goods or services. An assessment by the Joint United Nations Programme on HIV/AIDS (UNAIDS) and the Global Fund to Fight AIDS, Tuberculosis, and Malaria of the availability and quality of subnational data on sexually transmitted infection (STI) or HIV prevalence, behaviors, and coverage of HIV testing and size estimates for key populations in low- and middle-income countries between 2001 and 2015 found wide variation in the definition of key populations and few examples where definitions were sufficiently consistent to allow trend analysis [11].

Second, as progress is made in reaching the most accessible members of key populations, the HIV epidemic will reach a phase [12] where transmission becomes more concentrated in subgroups that are more hidden, mobile, and harder to reach. Tracking the epidemic among these dynamic subgroups and monitoring program coverage and outreach to them will require an SI system that is flexible, able to identify the emergence of subgroups, and align surveillance trends with program coverage trends. Bio-behavioral surveys such as those recommended by the UNAIDS and WHO Working Group on Global HIV/AIDS and STI Surveillance, currently operate on a 2- to 4-year cycle and, thus, will be challenged to provide flexibility and timely responses to program needs for information. If the coverage area for the survey does not match the catchment area for the specific programs or clinic services, the value of the survey data for immediate local use may be further obscured.

Third, beyond the challenges of aligning population definitions, identifying subgroups, and geographic reach, there is the challenge of reconciling the different sources of bias in and across survey methods, program monitoring systems, and routine medical record data systems. Different survey and population size estimation methods can result in different HIV prevalence and size estimates for the same population in the same location [13]. Methods such as programmatic mapping rely on information directly obtained from members of the community of interest, as well as indirect information obtained about key populations indirectly from other people who are engaged with the community. This mixed-methods approach may provide information that differs from surveys of population members only. Venue-based estimates are not often adjusted to include individuals who do not attend venues. Additional biases can emerge when sampling does not take mobility of the population into account, or if stigma, fear, or safety concerns limit engagement with key populations at public venues. Estimates from respondent-driven sampling (RDS) may be biased if there is significant clustering by subgroup [14] or geographic area [15]. As more members of key populations are tested and linked to care, it is possible that those who already know they are living with HIV will disproportionately refuse participation in surveys, further biasing the prevalence estimates. Tracking the epidemic and assessing program coverage among persons who move in, out, or across one or more key populations over time [16,17] is challenging but important to monitor coverage among those who may be least likely to be reached. For example, a transgender woman who sells sex and injects drugs may be less likely to access harm reduction services where stigma against transgender women may exist.

Finally, although estimation of the number and percent of new HIV infections is one of the 10 global indicators recommended in the new guidelines, there are methodological and operational challenges of validly measuring incidence among key populations [3]. The most epidemiologically rigorous approach to measure incidence, a prospective cohort study, requires significant resources to develop and maintain, so, it is rarely feasible and can be difficult to interpret given limited generalizability or if the cohort is mobile with significant loss to follow-up [18]. Measuring the social, political, and economic drivers [19,20] of HIV incidence among key populations is also challenging, but efforts to prevent HIV transmission will be limited if we fail to acknowledge the role of these drivers or the structural interventions [20] designed to address them.

We reviewed the published history of HIV surveillance among key populations to put the current challenges into context; identify future opportunities to enhance the use of surveillance data, programmatic data, and medical record data; describe the HIV epidemic among key populations; and measure the coverage of HIV prevention, care, and treatment programs.

## Methods

We described the historical expansion of HIV surveillance using published guidelines from WHO and UNAIDS, early surveillance reports identified using MEDLINE, and coauthor recollection.

We described the current salient features of four sources of SI: national household surveys, targeted bio-behavioral surveys, medical record data, and program data sources. We included known strengths, weaknesses, and opportunities for improved use of these data sources, with a focus on estimating elements of the treatment and prevention cascades [21,22].

We also review published estimates of 90-90-90 indicators based on these data sources to identify the availability of cascade estimates for key populations and the extent to which they drew on program, survey, and treatment databases. We conducted a title or abstract search in MEDLINE using the terms: HIV AND [HIV Testing OR population size estimate OR Viral Suppression OR antiretroviral therapy] AND [key populations OR MSM OR sex workers OR injection drug use OR prison OR transgender OR concentrated epidemic] AND [program data OR surveillance data OR routine data OR medical records]. We included manuscripts based on two criteria: (1) it reported any elements of the 90-90-90 HIV treatment cascade (population size, the proportion of the population who know their HIV status, the proportion of the population with HIV that is receiving antiretroviral therapy [ART], and the proportion of the population that is achieving viral suppression) for key populations from low- or middle-income countries and (2) it described strengths or weaknesses of data sources, or issues of aligning data from different sources. For studies where inclusion criteria could not be determined based solely on the abstract, we searched the full article.

## Results

### History of Surveillance Among Key Populations

Surveillance of the HIV pandemic has evolved over the past three decades, but from the earliest days it has included information about key populations. Early clinical case reports of patients with AIDS in Haiti in 1983 [23], in the Democratic Republic of Congo (Zaire) in 1984 [24], and in Rwanda 1984 [25], focused on clinical manifestations and immunological findings but also noted the presence or absence of behavioral risk, including homosexuality, injecting drug use, and prostitution. In 1985, a human T-cell lymphocyte virus prevalence study in Thailand [26] identified specific high-risk groups as male, homosexual sex workers; thalassemia patients; female sex workers; parenteral drug users; male, venereal disease patients; and blood donors. In 1986, a cohort of gay men in a hepatitis B study was assessed for evidence of AIDS [27]. A *Lancet* report from Rwanda in 1985 conveyed the first clear recommendation to focus on the risk posed by sex work [28]. The report concluded that “Since prostitution is widespread in Central African cities...infection may exist in a large, unconfined group of the general heterosexual population...Among heterosexual populations, prostitutes and probably their male customers should be regarded as high-risk groups.”

Subsequently, guidance surrounding data collection and monitoring for high-risk subgroups started to appear. In 1985, a WHO Coordinating Center Report [29] noted that “An important aspect of WHO activities...will be the collection of data on the incidence of the disease or its causative virus by Member States and the WHO Collaborating Centers...Wherever possible, information on the gender, age, recognized risk factor (if any), and major clinical features should also be provided.” In 1989, the WHO unlinked anonymous testing (UAT) guidelines addressed the ethics of compulsory HIV testing by stating that compulsory testing is unethical and that UAT can only occur when blood was already being taken for another purpose [30]: Information, such as sex and exposure category (if known), may accompany the unlinked anonymous specimen, but the possibility of indirectly identifying people infected with HIV must be eliminated by ensuring that this information is not too discriminating, for example, an age group should be used rather than specific age in years [31]. Despite these guidelines, there is some evidence that sex workers have been subjected to compulsory testing [32,33], at least occasionally since the early years of the HIV epidemic, raising issues of security and confidentiality for key populations.

In 1999, WHO published a comprehensive guide outlining the specific data elements required for STI case reporting in clinical settings [34]. Core elements of this guide included diagnosis, reporting site, date of visit, gender, age group, age, or date of birth. Optional data elements included residence, low education or socioeconomic status, clinical syndrome, anatomic site of infection, date of symptom onset, risk behaviors, pregnancy, history of STI, and treatment. Suggested indicators of risk behavior included the number of sex partners in the past 90 days (or 12 months), whether there were any new sex partners in the past 90 days, the gender of sex partners in the past 12 months (or their sexual orientation), condom use during the last sexual intercourse, drug use in the past 12 months, and giving or receiving money or drugs for sex in the past 12 months.

In 2000, WHO published guidelines for second generation surveillance [35], marking the first description of the strategy to conduct surveillance among antenatal clients in countries with generalized epidemics and among high-risk groups (including sex workers and MSM) among countries with concentrated and low-level epidemics. Second generation surveillance expanded the objectives of surveillance beyond HIV prevalence to include behavioral surveillance and AIDS case reporting. Population-specific questionnaire modules and indicators were developed.

In 2013, these guidelines were updated to incorporate the experiences of countries implementing second generation surveillance over the past 10 years and to incorporate changes in survey methods and laboratory diagnostics [36]. Although guidelines note differing objectives for surveillance (to track how the epidemic in a country is changing) and monitoring and evaluation (to track how effectively programs are responding to the epidemic and whether the outcomes and outputs correspond to the activities planned), the guidelines recommended that the systems be designed to be complementary. Surveillance and survey outcome and impact data should be used to assess the national program response. Program data should provide inputs, outputs, and outcomes to the national monitoring and evaluation system.

In response to each set of guidelines, surveys and surveillance systems were dynamically changed, though the implementation varied by country, region, epidemic profile, and study objective. Currently, many countries have yet to achieve the recommendations from 2103 to align program and surveillance data to describe the HIV epidemic and evaluate the response. Alignment with health record data is a further challenge. Surveillance activities are often implemented with little regard for clinical programs, partly because HIV status and ART were considered too confidential to ask about in surveys. Clinical data can be challenging to triangulate with survey data because definitions of geography or reference period and population do not necessarily align. The current recommendation to estimate the HIV treatment cascade represents a paradigm shift in surveillance and program monitoring because valid estimates of cascade indicators for a district require alignment of definitions, geography, and reference period across survey, program, and treatment databases.

### Data From Bio-Behavioral Surveys and Programmatic Mapping

Bio-behavioral surveys of key populations have been the backbone of HIV surveillance for key populations over the past 15 years, particularly for HIV prevalence estimates and more recently for size estimates. They provide probability surveys that facilitate representative estimates. Survey data are used for reporting country-specific indicators to UNAIDS; for use in mathematical models, including spectrum estimates; funding requests; and to guide country-level program reviews. Survey instruments have varied by country but commonly include HIV prevalence, knowledge of HIV transmission routes, sexual behavior (including condom use), and information to estimate the size of populations. Knowledge of HIV status, ART status, and indicators of viral suppression are increasingly included in survey instruments.

Bio-behavioral surveys have strengths relative to health or program data to estimate HIV prevalence and the size of the HIV population. Properly designed and executed bio-behavioral surveys aim to obtain a probability sample of the population in contrast to health sector or program data, which have data only from clients using their services. Surveys can provide an independent evidence-based assessment of gaps in coverage for programs and health facilities and identify emerging epidemics [37,38]. Moreover, validated survey modules on HIV stigma, the accessibility of health services, violence, and sexual behavior can provide more in-depth information on sensitive topics and allow analysis to identify and explore associations between HIV infection and barriers to accessing prevention and other services to guide the implementation of programs. Finally, survey data can provide a profile of those who do not access services, those who are living with HIV but do not know their HIV status, and the profile of those initially linked to treatment who report that they have stopped treatment.

Bio-behavioral surveys, however, have limitations. They are expensive and time-consuming to implement well, including effective engagement of stakeholders. Well-conducted surveys require formative research to guide protocol development, care in translation and back translation, ethical review by the appropriate organizations, interviewer training, ongoing monitoring of data quality, recording of deviations from the protocol, strategies to ensure data confidentiality and protection of participants, and strategies to provide participants with test results and linkage to care if indicated. Because HIV surveillance often takes place outside the health care system and involves contracting an outside implementing institution, there must be careful collaboration between the survey team and those in the health care system to ensure linkage to care for those who test positive as part of the survey.

Although survey design may minimize the effects of selection bias relative to clinical and program data, the effects of self-presentation bias on the validity of self-reported data may be considerable [39,40]. The extent to which anal sex, commercial sex, multiple sexual partnerships, injecting drug use, unprotected sex (sex without a condom), and lack of adherence to ART are underreported is unknown. It is likely that there are shifts in the level of stigma associated with different behaviors that could affect the interpretation of trends from surveillance data. For example, the increased availability of ART has probably led to increased willingness to self-report HIV infection. Legalization of gay marriage may lead to increased willingness to report same sex relationships; crackdowns on MSM may have the reverse effect.

With support from the Global Fund and other donors, countries are using programmatic mapping to identify where to reach key populations and to estimate the size of key populations [41]. Programmatic mapping systematically surveys community informants in a defined geographic area to identify high-risk venues (also known as hotspots) where key populations can be reached. In addition to venue-level data, programmatic mapping can include surveys of a representative sample of venue patrons and workers and oversampling of key populations [42]. Various methods exist for programmatic mapping, but all of them share some strengths and limitations. Strengths for programmatic mapping include the programmatic value of the maps for locating sites where key populations can be reached. Size estimates can be calculated from programmatic mapping and the estimates used to plan outreach visits by peer educators. Limitations for programmatic mapping include the limitations common to other surveys of key populations (as above), as well as the bias arising from the fact that key populations who do not visit these venues and will be missed. Other limitations include the labor-intensive protocol required to ensure that all sites have been listed and a sufficient sample visited.

There are several opportunities for improving the value of surveys for program improvement: (1) aligning size estimates from surveys with program catchment areas; (2) characterizing those reached by the survey, but missed by programs; (3) using the Internet for recruitment of survey participants; (4) measuring the 90-90-90 cascade, including viral suppression; (5) characterizing subgroups [43], including measures of HIV incidence, prevalence, and program coverage; (6) measuring gaps in service delivery; and (7) measuring stigma and its association with access to and use of services. Use of standard stigma indicators in surveys, programs, and health sectors could facilitate improving the quality of care and retention in care and programs.

For example, providing a cluster of differentiation 4 count at the time of the survey in Malawi to female sex workers living with HIV improved the acceptability of HIV testing and facilitated collecting an additional blood sample to estimate the proportion of female sex workers who had achieved viral suppression [44]. Another promising method to identify new HIV infections among key populations is phylodynamic analyses [45,46]. Additional strategies to improve the value of surveys to programs include better engagement of program participants in the design, implementation, and analysis of survey data, including contribution of questions related to specific program elements, as consultants in readiness assessments, as social mobilizers for recruitment of key populations, and as interpreters of the data in data-use workshops**.** In addition, if available, coverage maps from survey data could be provided to programs and workshops held to compare indicators from survey with indicators obtained from programs.

### Data From Medical Records

Routinely collected data from medical records and case-based surveillance systems have recently become a focus of development to facilitate measuring progress along the cascade. Where they exist, these data provide the number of persons on treatment. More sophisticated systems monitor progress along the cascade at the individual level from the first positive HIV test to viral suppression. Advocates argue that after HIV diagnosis, all cascade indicators recommended in the new Consolidated Guidelines for HIV Surveillance [47] could be estimated from case-based surveillance [48]. There is the hope that as these systems become routine and new technologies emerge to link data systems, surveillance will improve from the current cross-sectional approach to an ongoing longitudinal dynamic system that can more accurately identify those lost to follow-up.

Challenges in using case-based surveillance and other analyses of medical records for measuring the cascade among key populations in resource-constrained countries are evident already. The system requirements for tracking individual medical records are not available in many settings. In addition to the usual issues associated with solving the problem of deduplicating reports from various sources (eg, interoperability of computer systems, lack of standardization across providers for reporting, lack of timeliness in reporting, and lack of a unique identifier, UID, protocol), a successful case-based surveillance system to monitor the cascade among key populations would require an indicator of key population status in the surveillance record. Inclusion of this indicator is problematic. People may not self-identify as a member of a key population or want the indicator on their medical record [49,50]. Current guidelines do not recommend collecting risk behaviors, that is, key population status, in medical records if it is not clinically relevant. Discriminatory behavior can lead to dangerous situations for members of the populations; in fact, records from MSM services were recently seized by police in Tanzania.

In addition, other factors pose challenges for the unique identification of key population members. It is not unusual for sex workers or gay men to adopt a second identity to hide their affiliation and present at different clinics based on identity. Defining membership in a key population may be differently interpreted across facilities. Membership in a key population may be quite dynamic, causing problems for interpretation of the cascade over time. Biometric UIDs such as fingerprint scans facilitate monitoring at the individual level across data sources but require careful introduction into the community, technical support, data protection schemes, ethical review, and ongoing monitoring.

Due to the stigma associated with being a member of a key population and the lack of a key population identifier in the record, it is likely that case-based surveillance systems will underestimate the size of key populations. Health sector data will overestimate prevalence if those who are infected are more likely to seek clinical care (Berkson’s bias) or if clinics with a higher prevalence of infection among patients are selected for inclusion in surveillance [51], but could underestimate HIV prevalence if clinicians are less likely to directly indicate HIV status in the patient’s medical record (unacceptable disease bias) [52].

Some promising methods, however, are emerging to improve linkages between data sources when UIDs are not available, or an identifier fails to uniquely identify persons. One approach being piloted in the Dominican Republic is a follow-up survey of a sample of persons living with HIV in the treatment database to determine key population membership so that the cascade can be estimated for this subset of persons in the database. Improved probabilistic matching strategies based on available data such as name and birthday may be able to link an individual’s records across multiple programs when UIDs are not available. A MEDLINE search in January 2017 of articles related to probabilistic matching of medical records revealed 68 articles on the topic of probabilistic matching of medical records for HIV, of which 24 were published in the past 5 years. Free computer programs to improve deduplication of records are available, and efforts to evaluate the validity of probabilistic matching have been conducted [53].

Finally, new analytic tools are being developed to provide longitudinal measures of the cascade from treatment databases. These cascades indicate the time spent on the pathway from first positive HIV test to reaching viral suppression, the last 90 in the treatment cascade. The longitudinal HIV care and treatment cascade provides an estimate of the person time spent in each of the compartments of the HIV care continuum [54]. In summary, opportunities exist to leverage service delivery data to help both individuals and programs address issues for key populations. There remains much work to improve the quality of these data; survey data will remain of significant value for the foreseeable future; active surveillance from targeted surveys.

### Opportunities: Program Data From Key Population Programs

Since the early 1990s, there has been an acknowledgment that key populations are at greater risk of acquiring and transmitting HIV, are less likely to obtain services, and require specific services. Nongovernmental organizations or special outreach programs operating from government clinics may provide more acceptable and tailored services for key populations and may be more willing to engage key populations in target setting, advocacy, and addressing barriers to uptake of services. Some mature programs such as the Avahan sex worker interventions in India [55] collect longitudinal data on at risk populations, conduct size estimates, track intervention coverage, and track HIV prevalence. These programs illustrate that under certain conditions, with adequate resources, leadership, and stable funding, mature programs can set targets based on program data, routinely assess whether targets are met, and only minimally rely on independent HIV surveillance surveys.

Some of the challenges of using program data revolve around the variable quality of program data, arising partially from the broad array of data collection strategies, training, and available support. The main challenge is that program data is not readily generalizable to the entire key population (selection bias), as those who do not visit programs are likely to be different than those who do. Latecomers to programs differ from the early volunteers; thus, the maturity of a program will affect the risk profile of its participants. Even an umbrella program with multiple service delivery sites that employs a UID may comprise an unstable cohort if there is significant mobility across programs and loss to follow-up. Recruitment, attrition, and reach are often not measured systematically within programs even if the capacity to do so theoretically exists [53]. New guidelines for the development, use, and expansion of UIDS for key populations describe some of the technical and ethical challenges in linking program data [56].

### National Household Surveys

Probability samples of national household surveys such as the Demographic and Health Surveys (DHS), the AIDS Indicator Surveys, and the newer Population-Based HIV Impact Assessment provide insight into the geographic distribution of HIV across a country but often fail to provide much insight into the HIV epidemic among key populations [57]. Members of key populations may be missed by these surveys for several reasons: they may not be members of a household if they are homeless, resident in brothels or guesthouses, or in school; they may not report [40] sex work, injecting drug use, or male-with-male sex; and they may be more mobile and less likely to be available for an in-home interview. One assessment [58] of the mobility among 1653 female sex workers in Johannesburg, Rustenburg, and Cape Town, South Africa, found that 85% were migrants (39% internal and 46% cross-border). Key populations rarely comprise more than 3% to 8% of the general population. Financial and logistics constraints usually preclude interviewing a sufficient number of people in a household survey to provide an adequate sample of any key population.

Going forward, however, there may be opportunities for greater use of national household survey data. A new incidence model drawing on DHS data estimates the distribution of new infections in a population for groups, including key populations [18]. Estimates of the number of sex workers in the Dominican Republic were also estimated by Bayesian models extrapolating size estimates from known areas to areas without estimates using national survey data available in all areas [59]. The network scale-up method [60,61] has had success with modules added to some household surveys to estimate the size of key populations.

### Literature Review: Estimates of the 90-90-90 Treatment Cascade Among Key Populations

We identified 14 publications where at least one of the 90-90-90 indicators was estimated. Three provided estimation of each indicator. The first was an RDS survey among MSM in Moscow, Russia using RDS [62]; the second estimated 90-90-90 indicators using mathematical modeling based on inputs from both survey and programmatic data [63]; and the third was an abstract about MSM and people who inject drugs) in India [64]. Three studies only estimated the first 90 [38,65,66] and four only the second 90 [67-70]. Two studies estimated the first and second 90 [71,72], and three survey-based studies estimated all three indicators of 90-90-90. Among the three studies identified that included estimates of indicators of 90-90-90 from program data, two estimated the second 90 and third 90 [73,74], and one estimated the first 90 and second 90 [75]. In the two studies identified with estimates based in medical record data, only the second 90 was estimated from an ART database [76,77].

## Discussion

### Principal Findings

Current reliance on active data collection processes, including key population–specific surveillance surveys, is warranted both to collect specific critical information that cannot be obtained from service or other program data and to provide a representative depiction of the HIV epidemic and response. Elements of the latter may be replaced in the future by passively collected routine program and medical record data for key populations. Even in mature programs with years of investment in reaching and treating key populations, national programs must leverage data from program data, medical records, and surveys, as any single source will be insufficient to understand the HIV epidemic, monitor care, and track progress in prevention and along the treatment cascade. Bio-behavioral surveys, although expensive, have proven successful in measuring gaps in program coverage that are not yet revealed by program or medical record data. Improving methods to estimate the treatment cascade from medical records and maintain data security and patient confidentiality will remain a high priority. Strategies to gain insight from multiple sources will require efforts to align geographic catchment areas, definitions, subgroups, and indicators.

### Limitations

There are several limitations of this analysis. Important events in the history of surveillance were omitted for brevity. An exhaustive account of the global history of HIV surveillance in the context of key populations is out of scope; however, an overview of the progress and challenges were important to provide some context for the WHO SI guidelines. Important issues regarding measurement of community engagement, data quality, data use, mobility, and the effects of interventions on HIV transmission could not be addressed sufficiently. We did not describe the many size estimation methods available or address issues regarding the validity of these size estimation methods. For transparency, we recommend reporting the methods used to estimate population size and construct denominators for 90-90-90 estimates [11].

Although most peer-reviewed studies identified restricted the analytic sample to highlight a specific key population group, these analyses often included details about additional high-risk behaviors, documenting overlap in population membership. Due to the different risk behaviors of key population subgroups, the overlap between and among groups, and the variety of legal restrictions across countries, the opportunities and challenges related to disaggregating and aligning surveillance data, programmatic data, and medical record data are considerable.

Currently, complete estimates of indicators for the size of key population groups and estimates for indicators of the 90-90-90 targets are not generally available. There has been a movement to increase the use of programmatic data to inform the HIV epidemic among key populations, although evidence of the quality and validity of estimates from these data are lacking [16,78,79,80]. Targeted programs only reach a small fraction of key populations in most countries. Program data often overestimate HIV prevalence and underestimate the size of key populations, possibly because people who are infected are drawn to programs, whereas others avoid it. Size estimates can also be overestimated because of incentives at the program level or individual peer recruiter level if size estimates are larger. Critics suggest that inflated size estimates lead to inflated program targets that are impossible to meet.

Using programmatic data or medical record data for reporting disaggregated estimates of the 90-90-90 targets is particularly challenging when high-risk behaviors are overlapping in key populations, and programs address just one risk behavior. All individuals at risk of HIV acquisition and transmission may not identify as a member of the benefactor population [9,10]. There are programmatic data, particularly from southern and eastern Africa, showing young MSM disproportionately access interventions compared with older MSM. The older MSM are often in relationships with women, and they have heightened fears that their sexual attraction to men may be inadvertently disclosed [81,82]. These older men are uncounted, with high HIV burden, and without targeted services. Collecting robust, high-quality monitoring, evaluation, and surveillance data from programs to estimate the size of populations at highest risk is challenging even when resources are plentiful [55]. Given the challenges in constructing a denominator from programmatic and medical record data, care continua and estimates of 90-90-90 from different data sources are biased or fragmented.

UIDs may offer a solution to linking individuals across surveys, programs, and health care settings if the ethical, logistic, and technological challenges of implementing UIDs for marginalized and criminalized populations can be resolved. In many cases, especially when injection drug use, sex work, or homosexuality is illegal, providing details about high-risk behaviors and linking such information to a permanent medical record is a risk that leaves these already marginalized populations more vulnerable. WHO’s new case reporting and patient monitoring guidelines specifically do not include risk behaviors in the patient monitoring data forms because of the potential for harm to patients from stigmatized populations [83]. These details are therefore often underreported [69,76,84]. Other promising approaches include using the Internet to reach key populations who engage in online community groups.

In conclusion, we recommend ongoing engagement with key population communities in the improvement and alignment of SI indicators across current data sources and exploration of new sources of data. The goal of SI is to improve the adequacy, acceptability, safety, and effectiveness of the public health response to the HIV epidemic among these populations.

## Abbreviations

ART: antiretroviral therapy
DHS: Demographic and Health Surveys
MSM: men who have sex with men
RDS: respondent-driven sampling
SI: strategic information
STI: sexually transmitted infection
UAT: unlinked anonymous testing
UID: unique identifier
UNAIDS: Joint United Nations Programme on HIV/AIDS
WHO: World Health Organization
